# Transparency in authors’ contributions and responsibilities to promote integrity in scientific publication

**DOI:** 10.1073/pnas.1715374115

**Published:** 2018-02-27

**Authors:** Marcia K. McNutt, Monica Bradford, Jeffrey M. Drazen, Brooks Hanson, Bob Howard, Kathleen Hall Jamieson, Véronique Kiermer, Emilie Marcus, Barbara Kline Pope, Randy Schekman, Sowmya Swaminathan, Peter J. Stang, Inder M. Verma

**Affiliations:** ^a^National Academy of Sciences, Washington, DC 20001;; ^b^Science Journals, American Association for the Advancement of Science, Washington, DC 20005;; ^c^New England Journal of Medicine, Boston, MA 02115;; ^d^American Geophysical Union, Washington, DC 20009;; ^e^SAGE Publishing, Thousand Oaks, CA 91320;; ^f^Annenberg School for Communication, University of Pennsylvania, Philadelphia, PA 19104;; ^g^PLOS (Public Library of Science), San Francisco, CA 94111;; ^h^Cell Press, Cambridge, MA 02139;; ^i^The National Academies of Sciences, Engineering, and Medicine, Washington, DC 20418;; ^j^University of California, Berkeley, CA 94720;; ^k^eLife, Cambridge CB2 1JB, United Kingdom;; ^l^Nature Research, San Francisco, CA 94104;; ^m^University of Utah, Salt Lake City, UT 84112;; ^n^Salk Institute for Biological Studies, San Diego, CA 92037

**Keywords:** authorship principles, research transparency, scientific integrity

## Abstract

In keeping with the growing movement in scientific publishing toward transparency in data and methods, we propose changes to journal authorship policies and procedures to provide insight into which author is responsible for which contributions, better assurance that the list is complete, and clearly articulated standards to justify earning authorship credit. To accomplish these goals, we recommend that journals adopt common and transparent standards for authorship, outline responsibilities for corresponding authors, adopt the Contributor Roles Taxonomy (CRediT) (docs.casrai.org/CRediT) methodology for attributing contributions, include this information in article metadata, and require authors to use the ORCID persistent digital identifier (https://orcid.org). Additionally, we recommend that universities and research institutions articulate expectations about author roles and responsibilities to provide a point of common understanding for discussion of authorship across research teams. Furthermore, we propose that funding agencies adopt the ORCID identifier and accept the CRediT taxonomy. We encourage scientific societies to further authorship transparency by signing on to these recommendations and promoting them through their meetings and publications programs.

## Integrity of Authorship

Publications are an important measure of research productivity for scientists across all disciplines, so much so that the quest for authorship status invites gaming the system in ways detrimental to both scientists and the research enterprise. Calls for reform of the authorship system have gone unheeded (1), perhaps for lack of specific, actionable plans. The movement to replace or augment publications with broader measures of scientific productivity is still in its infancy.

The notion of authorship implies both credit and accountability (2). However, authorship conventions vary across disciplines (3, 4), across cultures internationally (5), and even between research groups and laboratories in the same discipline. The various conventions differ in their expectations of what effort earns authorship, what the order of authorship signifies (if anything), how much accountability for the research the corresponding author assumes, and the extent to which authors are accountable for aspects of the work that they did not personally conduct. We propose an actionable plan that consists of a set of journal policies to remove ambiguity in expectation for authors and ongoing university stakeholder meetings for managing the cultural and disciplinary variability in deciding who has earned authorship.

## Recommendations for Journals

Several authorship practices deemed detrimental to research (6–8) and solutions implementable by journals are included in Table 1. These recommended solutions and others to clarify responsibilities of authorship are codified in the following draft journal policies and procedures, many of which are already best practices in leading scientific journals.

**Table 1.** Recommendations for journals

Detrimental authorship practice	Definition	Proposed solutions
Ghost authorship (7)	Authors who contributed to the work but are not listed, generally to hide a conflict of interest from editors, reviewers, and readers.	Corresponding author must confirm that all who deserve authorship are listed; conflict of interest declarations; ethics training in collaboration with universities/research institutions.
Guest/gift/honorific authorship (8)	Individuals given authorship credit who have not contributed in any substantive way to the research but are added to the author list by virtue of their stature in the organization.	Journals require each author have a transparent, identified, legitimate role in the research.
Orphan authorship	Authors who contributed materially to the work but are omitted from the author list unfairly by the drafting team.	Corresponding author must confirm that all who deserve authorship are listed; ethics training in collaboration with universities/research institutions.
Forged authorship	Unwitting authors who had no part in the work but whose names are appended to the paper without their knowledge to increase the likelihood of publication.	Journal contacts all authors to confirm they acknowledge their contribution to the work.

### Set Standards for Authorship.

As the first step, we recommend that journals adopt the following statement as a best practice for crediting all authors of a paper:

Each author is expected to have made substantial contributions to the conception or design of the work; or the acquisition, analysis, or interpretation of data; or the creation of new software used in the work; or have drafted the work or substantively revised it; AND to have approved the submitted version (and any substantially modified version that involves the author’s contribution to the study); AND to have agreed both to be personally accountable for the author’s own contributions and to ensure that questions related to the accuracy or integrity of any part of the work, even ones in which the author was not personally involved, are appropriately investigated, resolved, and the resolution documented in the literature.

This statement is adapted from a similar one developed by the International Committee of Medical Journal Editors that is widely used within the medical publishing community. The statement has been generalized to encourage broader adoption.

### Provide Expectations for Corresponding Authors.

As a second step, journals should clearly articulate their expectations for corresponding authors (CAs). Appropriate roles for the CAs are as follows: ensuring that all listed authors have approved the manuscript before submission and that all authors receive the submission and all substantive correspondence with editors, as well as the full reviews, verifying that all data, materials (including reagents), and code, even those developed or provided by other authors, comply with the transparency and reproducibility standards of both the field and journal. This responsibility includes but is not limited to: (*i*) ensuring that original data/materials/code upon which the submission is based are preserved following best practices in the field so that they are retrievable for reanalysis; (*ii*) confirming that data/materials/code presentation accurately reflects the original; and (*iii*) foreseeing and minimizing obstacles to the sharing of data/materials/code described in the work. The CA should be responsible for managing these requirements across the author group and ensuring that the entire author group is fully aware of and in compliance with best practices in the discipline of publication.

To discourage ghost authorship, CAs must reveal as appropriate whether the manuscript benefited from the use of editorial services that, if unacknowledged, might constitute an undisclosed conflict of interest. Examples include use of an editor from an organization that may have a vested interest in slanting the results or reliance on a technical writer at a level that would warrant authorship credit. These situations might variously be addressed by including a statement in the acknowledgments, by describing the effort in the methods section, or by adding an author. Many journals require CAs to indicate whether any authors on earlier versions have been removed or new authors added and why. This simple step discourages the practice of guest authors or orphan authors. It is incumbent on the CA to ensure that all authors (or group/laboratory leaders in large collaborations) have certified the author list and contribution description: that all authors who deserve to be credited on the manuscript are indeed identified, that no authors are listed who do not deserve authorship credit, and that author contributions, where they are provided, are expressed accurately.

### Commit to the Use of the Contributor Roles Taxonomy.

Our third recommendation is that journals commit to use and where appropriate, extend the CRediT (Contributor Roles Taxonomy) taxonomy (9) as, in our estimation, the best currently available method for embedding authors’ contributions in journal metadata. The 14 CRediT taxonomy categories for contributor roles are evidence-based in that they were distilled by a stakeholder group from free-form author statements and acknowledgments from research in the physical, life, and social sciences. When combined with ORCID identifiers (iDs), the potential exists to reliably link author records to publications, to capture author contributions in the journal’s metadata, and to track and retrieve an individual’s authorship contributions across publications and across time.

Uniformity of declarations across journals is in the authors’ best interests because such statements will not need to change if the same paper is rejected and submitted to another journal or if the same research team with similar roles submits a follow-up study to the same or another outlet. Moreover, confirming the authors’ contributions as the manuscript is finalized is likely to create a more accurate record of what transpired than asking participants to recall the information post facto (e.g., when authors are considered for promotion, research grants, or major prizes).

The CRediT taxonomy’s standardized vocabulary and consistent framework should facilitate authorship discussions among contributors to a study. In this regard, it is important to note that the taxonomy includes but is not limited to traditional authorship roles. It is intended to describe authors’ contributions within the framework of authorship standards, not to define such standards. Adopting the CRediT taxonomy would also help alleviate some of the confusion across disciplines and cultures regarding the meaning of author order (10). These differences are currently so ingrained that forcing a common standard is impractical and will be unnecessary if the CRediT taxonomy becomes widely adopted.

We recommend that the CRediT taxonomy roles be embedded within author metadata rather than solely as a separate paragraph of text, often linked only to author initials. Journals’ adoption of the CRediT taxonomy in both human- and machine-readable forms, as part of the publication and its metadata, will facilitate transparency of author contributions in different contexts, via syndication, indexing, and abstracting services, and possibly future applications across journals. We recognize that full implementation will require some further development of common schemas and standards across journals, organizations, and communities.

A handful of journals currently use the CRediT taxonomy, and we realize that adoption by all journals is an aspirational goal. However, now is the time, in the early phases of journal experimentation with attributing contributions to authors, to champion this standard and its widespread adoption. Use of the CRediT taxonomy need not be the only way for a journal to capture how each author earned the right to be listed [e.g., additional information, including further details on roles and contributions, can be captured in the acknowledgments (for an example of specifics in author contributions, see ref. 11) or footnotes or a separate statement], but if CRediT roles are available consistently, in machine-readable form and through the journal metadata, author contributions will transition from hearsay to quantifiable evidence.

### Require Authors to Adopt ORCID iDs and Other Standard Identifiers.

Finally, we recommend that all journals in the physical, life, and social sciences require that authors have an ORCID iD and include it in the author metadata and all article presentations. To eliminate name confusion and ensure appropriate attribution of publications and citations to the correct authors, many journals are already requiring ORCID iDs for first, corresponding, or all authors. Although it cannot alone guarantee secure identity, adoption of ORCID iDs is one more check against author identity fraud.

Other persistent identifiers (PIDs) have emerged to uniquely identify different funders of research, and a multistakeholder collaboration is ongoing to establish standard PIDs for institutions. At the same time, some fields have adopted PIDs for samples. Development is under way to explore or extend PIDs for repositories and instruments that would lead to further transparency and integrity. We encourage journals not only to follow these developments but also to prepare for wider adoption when standards emerge.

## Recommendations for Research Institutions, Funders, and Societies

### Universities/Research Institutions.

Journals are ill-equipped to mediate authorship disputes or set expectations for authorship within project groups or teams. Universities and other research institutions should develop, post, distribute, and regularly review and update their policies on authorship. This process should actively involve faculty/investigators, postdoctoral scholars, graduate and undergraduate students, staff, and any others making important contributions to scholarship in the sciences. The same researchers should engage in open conversations at least annually to familiarize new employees (including foreign visitors) with the norms of the field. Questions answered by these policies would include how the organization or a research team within it decides which contributions warrant first authorship, coauthorship, acknowledgments, or no mention. The value of early discussion of authorship has been demonstrated (12). Because these decisions vary with the culture of various disciplines and nations, policies may vary across institutions and departments, but should not vary within a department. Transparency in how the decision is made before the research is undertaken can avoid later conflicts. To ensure that the same criteria for authorship are applied to personnel from different laboratories, universities should expect principal investigators involved in multilaboratory collaborations (even if across universities) to engage in the same discussion. When researchers or technical personnel make the same level and kind of contribution to a collaborative study, their status as author should not depend on the laboratory with which the person is affiliated.

### Funding Agencies.

Were they to adopt ORCID iDs as the preferred/default system for their principal investigators, funding agencies could easily tip the scales toward widespread acceptance of a truly international standard for uniquely identifying scholars. If every investigator supported by United States and international funding agencies was required to use an ORCID iD, the standard would become the norm. A positive step in this direction was the recent decision by the European Commission to recommend the adoption of ORCID iDs as its preferred system of unique identifiers for its ninth Framework Program (13). Another advancement is the recent announcement of the partnership between the NIH and ORCID to reduce burdensome multiple entries of data and to improve impact tracking (14).

Additionally, it would be helpful if in requests for bibliographical references in support of an application as well as in progress reports, funding agencies were to accept—or better yet, require—that representation of the applicant’s contributions to the work be expressed via CRediT roles. By importing citation formats in more complete CRediT terms, it would make clear the applicant’s contribution to collaborative research. An added bonus for funding agencies would be the ability to mine the CRediT files for needed reviewer expertise (9).

In the near future, another role for funders may be to underwrite efforts to seamlessly integrate the CRediT taxonomy, ORCID iDs, journal metadata, and scholarly archives—including public archives—into the public record.

### Scientific Societies.

Through editorials in their journals, special sessions at their meetings, and policies in their own publishing programs, many scientific societies have strongly endorsed efforts to increase transparency. We suggest that they build on these initiatives by organizing special sessions on the topic of integrity in authorship at scientific meetings to discuss issues relevant to each society. Each organization should consider how the society can move briskly in its own journal publishing program to implement the recommendations in this paper.

## Further Steps

We recognize that the path we have outlined is a work in progress. While a leap forward in authorship transparency, our proposals leave a number of vexing issues unresolved. The CRediT taxonomy provides a summary or snapshot of contributor roles, but does not allow for additional detail on exactly what each author did, including microattributions for figures, models, or datasets (10). To address such questions, we encourage the creation of a cost-free, web-based tool that facilitates experimentation by publishers and authors about ways to increase transparency and accountability in authorship beyond what is currently possible with CRediT taxonomy. As long as the tool exports the minimal information required by a journal, in a format that the journal can receive, it could still be useful and follow the traditional workflow. Journals may decide to preserve this additional information in acknowledgments, footnotes, or by other means.

We also recognize that additional development is needed for export of CRediT roles to ORCID records. This step should be undertaken as soon as is practical.

## Conclusions

The National Academy of Sciences has created a TACS (Transparency in Author Contributions in Science) website at www.nasonline.org/about-nas/Transparency_Author_Contributions.html, where it will list those journals that commit to setting authorship standards, defining responsibilities for corresponding authors, requiring ORCID iDs, and adopting the CRediT taxonomy. The site will also include those funding agencies that adopt ORCID iDs and accept the CRediT taxonomy. Our goal is to use this TACS website not only as a mechanism to measure growing transparency in authorship, but also as a resource for sharing and exchanging best practices in authorship policies that can inform discussions at university and research laboratories and departments.
